# Impact of Health System Structures on Caries Prevalence Among Schoolchildren in Germany and Saudi Arabia

**DOI:** 10.3390/children13040440

**Published:** 2026-03-24

**Authors:** Yasser Mansour Aljafen, Christian Heinz Splieth, Julian Schmoeckel

**Affiliations:** 1 Zircon Dental Clinic, Buraidah 52387, Saudi Arabia; dr.yasserj@hotmail.com; 2 Department of Pediatric Dentistry, University Medicine Greifswald, 17475 Greifswald, Germany

**Keywords:** dental caries, oral health, schoolchildren, preventive dentistry, healthcare system

## Abstract

Background and Objectives: Dental caries remains one of the most prevalent chronic conditions among children worldwide, with considerable variation in burden shaped by differences in health-system organization and preventive program implementation. Some countries have reported notable progress through coordinated school-based programs and supportive policy environments, while others continue to face challenges despite extensive public health efforts. This study aims to explore how selected structural and programmatic elements within national health systems may be associated with childhood caries outcomes, using Germany and Saudi Arabia as illustrative contexts. Materials and Methods: A descriptive comparative secondary-data analysis combined with legal and policy mapping was conducted using published national oral health surveys, systematic reviews, and governmental reports. Caries indicators (dmft/DMFT) for children aged 6–7 and 12 years were extracted following WHO criteria. Health system organization, preventive program coverage, and policy enforcement mechanisms were mapped and critically reviewed. No new primary data were collected, and no inferential modeling was performed. Results: Germany has achieved substantial reductions in childhood caries prevalence through a legally mandated school-based prevention program, supported by individual prophylaxis covered by health insurance. This framework corresponds with low mean dmft (1.73) among 6–7-year-olds and mean DMFT (0.5) among 12-year-olds. By contrast, Saudi Arabia continues to report elevated caries rates despite substantial public-health investments, with mean dmft (5.0) and mean DMFT (3.5), with over 90% of children affected. Preventive initiatives in Saudi Arabia remain fragmented across sectors and lack a unified legal mandate. Conclusions: The findings suggest that structured governance, coordinated prevention strategies, and reliable monitoring systems may be associated with more favorable oral health outcomes. In the context of Saudi Arabia, the persistently high caries burden suggests that strengthening national legislation, implementing an interoperable digital surveillance system, and improving the consistency of school-based preventive programs could support more equitable and effective oral health delivery.

## 1. Introduction

Dental caries remains one of the most common chronic diseases affecting children worldwide and continue to pose significant public health challenges due to their impact on well-being, daily functioning, and quality of life [1]. Although many high-income countries have achieved substantial reductions in childhood caries over recent decades, marked disparities persist both between and within nations [2].

Germany is among the most successful examples of sustained caries reduction. National surveillance data show that children and adolescents in Germany have healthier teeth than ever before, with a mean dmft (1.7) among 6–7-year-olds and a mean DMFT (0.5) among 12-year-olds—the WHO reference age group for international comparisons [3,4,5,6]. These values classify Germany as a low-caries-prevalence country according to WHO criteria.

By contrast, dental caries remains a significant public health concern among children in Saudi Arabia. Despite multiple public health initiatives, the prevalence of untreated caries remains high, with approximately 80% of primary dentition affected (mean dmft of 5.0) and 70% of permanent dentition affected (mean DMFT of 3.5) [3]. According to the Saudi Ministry of Health, the caries prevalence is 96% among 6-year-olds and 93.7% among 12-year-olds, underscoring the severity of the burden among school-aged children [4].

Understanding these differences requires examining not only epidemiological trends but also the structural and organizational characteristics of national health systems. To enhance the effectiveness of caries prevention programs for schoolchildren in Saudi Arabia, it is essential to assess the structure of the healthcare system, evaluate the public oral health framework, review preventive program organization, and analyze epidemiological patterns. Many developed countries refine their healthcare and oral health strategies by comparing their systems with those of others to identify strengths and weaknesses, thereby improving public health outcomes [5].

Therefore, this study aims to analyze how health system structures, preventive program organization, and policy frameworks may be associated with differences in childhood caries prevalence between Germany and Saudi Arabia. By comparing healthcare models, public oral health programs, and preventive strategies in both countries, this study seeks to identify key structural factors that could support more effective caries prevention efforts in Saudi Arabia.

## 2. Materials and Methods

### 2.1. Study Design

This study employed a descriptive comparative secondary data analysis combined with legal and policy mapping to examine differences in dental caries patterns and oral-health system structures relevant to schoolchildren in Germany and Saudi Arabia. The analysis drew on published epidemiological studies, national oral health surveys, and governmental oral health reports. No new primary data were collected, and no inferential modeling was performed.

### 2.2. Study Population

Data on dental caries prevalence for children aged 6–7 years (primary dentition) and 12 years (permanent dentition) were extracted following WHO guidelines using the DMFT/dmft indices [6]. In Germany, data were obtained from the National Oral Health Surveys conducted by the German Association for Youth Dental Care (DAJ e.V.). For Saudi Arabia, relevant studies meeting WHO criteria and reporting on these age groups were identified through targeted literature searches in PubMed, Scopus and Google Scholar that adhered to WHO diagnostic criteria for reporting caries prevalence in children. This standardized selection approach supports comparability between countries, although full one-to-one equivalence cannot be achieved because of differences in the health system structures and data availability.

### 2.3. Inclusion and Exclusion Criteria

The approach to data identification differed between Germany and Saudi Arabia due to differences in the availability of national epidemiological data. For Germany, caries data were obtained primarily from the standardized national oral health surveys conducted by the German Association for Youth Dental Care (DAJ e.V.), which provide population-based estimates for the WHO reference age groups of 6–7 and 12 years. Because DAJ surveys have been available only since the early 1990s, earlier caries trends were identified through peer-reviewed epidemiological studies reporting caries levels in the former East and West Germany.

In contrast to Germany, Saudi Arabia does not have a unified national surveillance system reporting age specific caries indicator. Therefore, a targeted literature search was conducted in PubMed, Scopus, and Google Scholar using combinations of the terms “dental caries,” “dmft,” “DMFT,” “children,” “schoolchildren,” “Saudi Arabia,” “oral health survey,” and “epidemiology.” Titles and abstracts were screened, and full texts of potentially eligible studies were reviewed.

Eligibility criteria: Included studies were required to report caries prevalence or mean dmft/DMFT values for schoolchildren of any school-age range, and to adhere to the WHO diagnostic criteria for caries assessment. Although data were extracted for all school-age groups, the 6–7-year and 12-year reference ages were used specifically for cross-country comparison between Germany and Saudi Arabia. Studies focusing on non-school-age children or on specific clinical populations, including children with special healthcare needs or those undergoing orthodontic treatment, were excluded to maintain comparability with the general schoolchild population. For Saudi Arabia, caries experience and prevalence data were extracted from studies published between 1982 and 2022, and all extracted data were cross-checked for accuracy. For Germany, the analysis incorporated all available national and regional data sources, including DAJ survey cycles from 1994/95 to 2016 and earlier epidemiological studies dating back to 1973.

### 2.4. Comparative Framework

A structured comparative framework was used to examine the differences in health system organization, preventive program structures, and oral health policies between Germany and Saudi Arabia. The comparison focused on domains emphasized in the WHO oral health system guidance, including governance and system structure, organization and coverage of school-based preventive programs, availability of individual preventive services, and national monitoring and surveillance mechanisms [7,8,9,10]. This approach allowed for the identification of system-level features that may be associated with variations in childhood caries outcomes across the two countries.

### 2.5. Data Analysis

Descriptive statistics were used to compare mean caries experience (dmft/DMFT) between Germany and Saudi Arabia across the standardized reference age groups (6–7 and 12 years). Longitudinal patterns in caries prevalence were examined using historical data where available, with particular attention to trends associated with national preventive strategies [11]. A thematic comparative approach was applied to evaluate the differences in healthcare system structures, legal frameworks, examiner calibration practices, and data documentation processes. Interpretive emphasis was placed on contextual factors that influence oral health outcomes, while acknowledging limitations such as regional data variability and inconsistent methodological standards, particularly within the Saudi Arabia literature.

## 3. Results

The findings of this comparative analysis highlight clear differences in the production, reporting, and processing of epidemiological data on dental caries among school-aged children in Germany and Saudi Arabia.

### 3.1. Historical Caries Trends in Germany

Caries prevalence among children in Germany has shown substantial improvements since the 1970s. In West Germany, DMFT scores among 13–14-year-olds peaked at 8.8 before declining to 5.1 by the 1980s. In East Germany, DMFT values remained relatively stable at 3.3 during the same period, followed by a modest reduction to 4.3 by 1992. Between 1983 and 1989, a 42% decrease in caries rates was observed among West German adolescents [12].

### 3.2. Current Caries Trends in Germany

Germany has seen significant improvements in children’s oral health over the past few decades. According to the German Association for Youth Dental Care (DAJ e.V.) [11], the mean DMFT score for 12-year-olds decreased by approximately 60%, from 2.44 in 1994/95 to 0.98 in 2004. Meanwhile, dmft scores for 6–7-year-olds declined by 25%, from 2.89 to 2.16 over the same period, further dropping to 1.87 by 2009.

**Figure 1:**
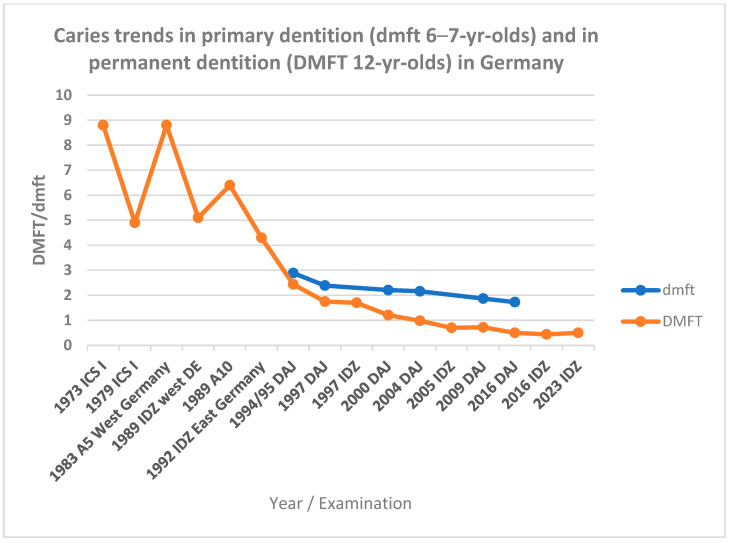
Trends in caries prevalence in Germany (1973–2023): Mean dmft scores for primary dentition in 6–7-yr-olds in first grade and DMFT scores for permanent dentition mostly in 12-yr-olds. Abbreviations: ICS = International Caries Surveillance; DAJ = German Association for Youth Dental Care; IDZ = Institute of German Dentists. Data sources: [12].

DAJ’s 2016 report indicates that caries-free dentition among 6–7-year-olds more than doubled—from 20% in 1995 to 42.6% in 2016. Among 12-year-olds, the proportion with naturally healthy teeth rose markedly from 15.3% to 75.8% over the same timeframe [11]. The Figure 1 illustrates these trends using a line chart, depicting the steady decline in DMFT/dmft scores across both dentitions from 1973 to 2023. Although the IDZ and DAJ studies employed differing recruitment methodologies for participants and examiners, their findings show a high degree of consistency across time points. The latest data point from DAJ (2016) reports a mean DMFT of 0.44 for 12-year-olds, based on a positively selected sample from sixth grade [11]. After statistical adjustment for educational background bias (≈15%) [13], this corrected DMFT aligns with the 2016 and 2023 IDZ benchmark of 0.5 DMFT [14], indicating stabilization in caries reduction among school-aged children in Germany with a low caries prevalence of ~20%.

Several federal states in Germany, such as Brandenburg and Thuringia, have established robust systems for collecting and publishing detailed caries data. These platforms provide annual updates based on compulsory dental examinations in kindergartens and schools, covering nearly all children up to 12 years of age and offering a comprehensive view of oral health trends. Although data collection was temporarily disrupted during the COVID-19 pandemic, the continuity and depth of these datasets remain exemplary and serve as valuable benchmarks for systemic evaluations and policy planning [11,13,14].

### 3.3. Historical Caries Trends in Saudi Arabia

In contrast to Germany’s standardized national surveillance system, Saudi Arabia lacks comprehensive population-wide data on dental caries prevalence. Most studies have been geographically limited, clinic-based, or age-specific, resulting in a fragmented epidemiological landscape and inconsistent indices across regions [3].

Despite these limitations, the available evidence spanning four decades consistently indicates high caries prevalence among Saudi children and adolescents. Early research conducted in 1982 reported a DMFT of 2.90 and 77.7% caries prevalence among 13–15-year-olds in Riyadh [15]. Through the 1990s, oral health surveys across five cities revealed DMFT scores ranging from 0.19 in 6-year-olds in Riyadh to 5.8 in 15-year-olds in Jeddah, with dmft values ranging from 2.1 in Rabagh to 6.4 in Madina [16].

Between 2000 and 2010, the observed DMFT rates varied significantly, from 0.16 in 6-year-olds in Riyadh to 7.35 in adolescents aged 15–19, while the dmft scores ranged from 2.68 in 12–13-year-olds to 7.77 in 5–7-year-olds in Tabuk [17].

**Figure 2:**
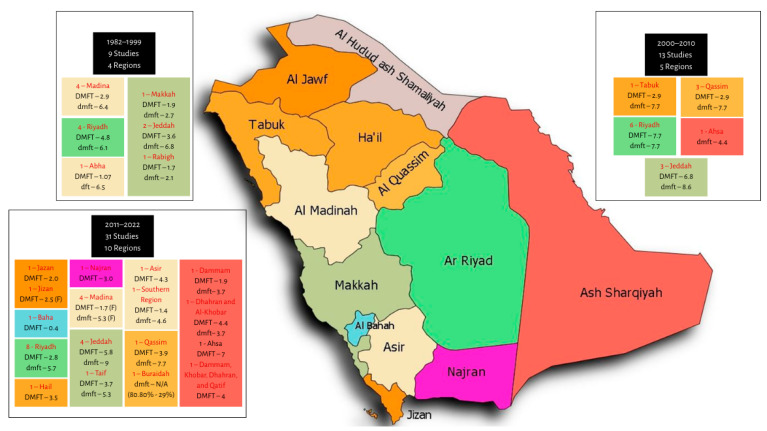
Geographical distribution of dental caries data across Saudi Arabia based on studies conducted between 1982 and 2022. Data sources: [15,16,17,18,19,20,21,22,23,24,25,26,27,28,29,30,31,32,33,34,35,36,37,38,39].

From 2011 to 2022, this variability persisted. Studies reported DMFT scores from 0.26 among 6–7-year-olds in Jeddah to 7.0 among 15–19-year-olds in Al-Ahsa and dmft scores ranging from 1.4 in 12-year-olds in Madina to a high value of 9.0 in 4–6-year-olds in Jeddah [18,19,20,21]. Figure 2 presents the distribution of reported caries values across Saudi regions from 1982 to 2022.

### 3.4. Legal and Policy Framework

Available national data from Germany, as reported in international analyses of the National Oral Health Survey [12,40,41] and data from the regional School Health Units in Qassim indicate that both Germany and Saudi Arabia have implemented school-based oral health programs targeting children at key developmental stages. Children aged 6–7 years (typically in first grade) and 12 years (typically in sixth grade) commonly receive preventive dental services in both countries. In addition, oral health examinations prior to school entry—conducted either during kindergarten or upon enrollment in first grade—are mandated by both German and Saudi health authorities.

#### 3.4.1. Children’s Oral Health Legislation and Policies

**Figure 3:**
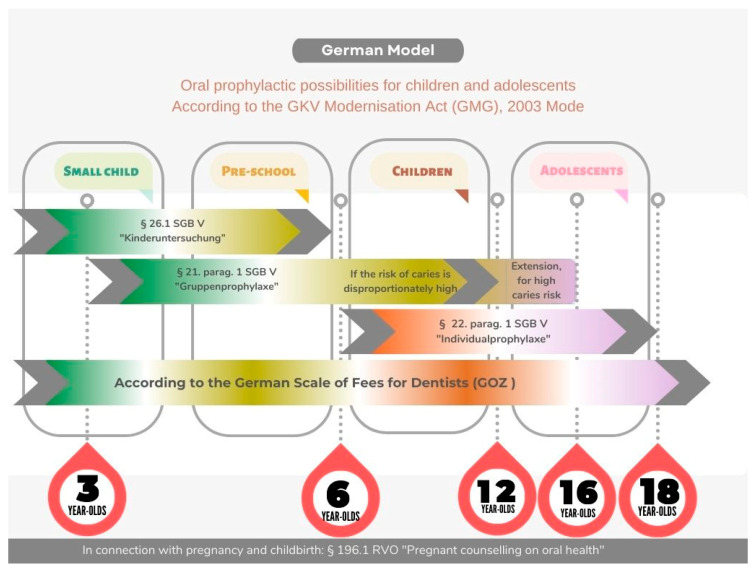
German oral prophylactic pathways for children and adolescents, based on age-specific entitlements outlined in Social Code Book V (SGB V) and the GKV Modernisation Act (GMG), 2003. Source: IDZ, 2004; Federal Law Gazette (BGBL) of 19 November 2003, p. 2190 [42].

Germany and Saudi Arabia differ substantially in the legal foundations and organizational structures governing children’s oral health. Germany operates under a legally codified preventive framework defined in the Social Code Book V (SGB V) [42], which mandates nationwide group and individual prophylaxis programs with age-specific entitlements. These include supervised brushing, fluoride varnish, fissure sealants, and structured early-detection examinations beginning in infancy. These entitlements are chronologically aligned with the child’s oral development, as illustrated in Figure 3, which maps Germany’s preventive services across key age intervals and corresponding SGB V provisions.

**Figure 4:**
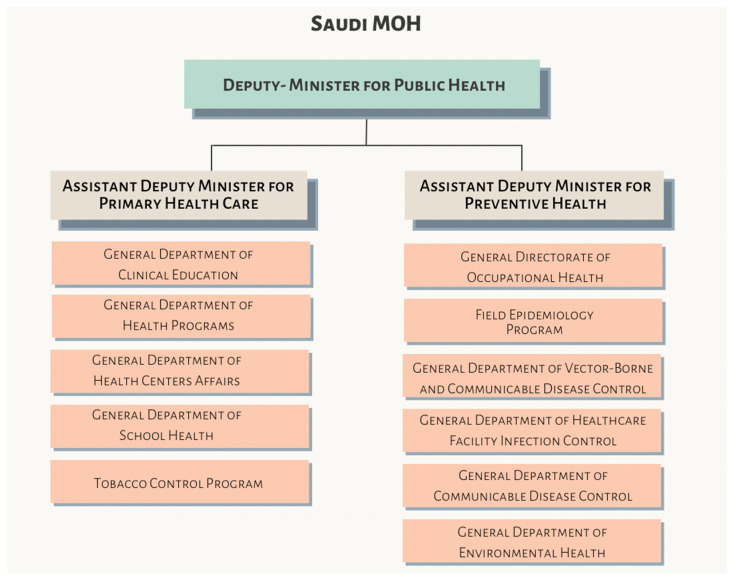
Saudi MOH primary health care and preventive health organization structure. Source: Saudi Ministry of Health website: https://www.moh.gov.sa/en/Ministry/About/Pages/Organizational-Structure.aspx (accessed on 5 February 2026).

**Figure 5:**
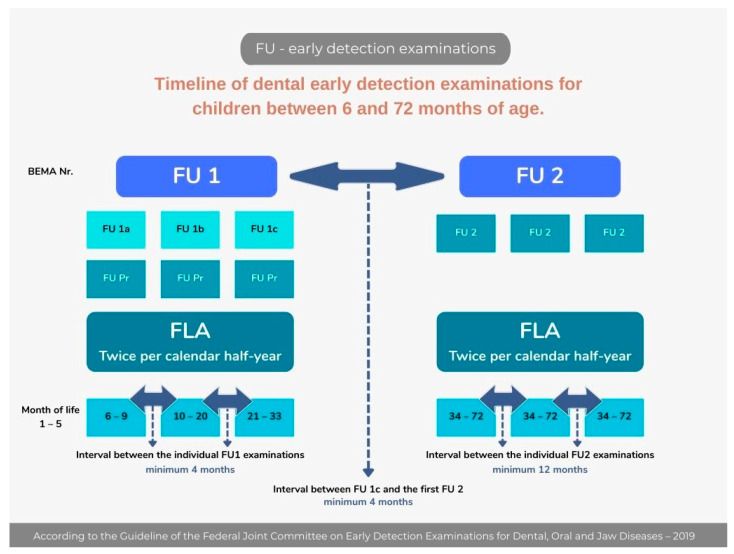
Timeline of early dental examinations for children between 6 and 72 months of age covered by statuary health insurance [43,44,45].

In Saudi Arabia, oral health services for children operate under general health provisions (Saudi Health Law Article 4) without equivalent statutory mandates for prophylaxis programs. Oversight is provided by the Ministry of Health’s Central Department of School Health, which delivers program-based services through regional Primary Health Care Centers (Saudi MOH Primary Health Care and Preventive Health Organization Structure, Figure 4). Programs such as the School Dental Prevention Program (SDPP) and the National Initiative for Preventing Dental Caries (NIPDC) provide fluoride application and screenings but function under programmatic rather than legislative frameworks. Germany additionally mandates early-detection examinations for children aged 6–72 months covered by public health insurance, as illustrated in Figure 5.

Table 1 summarizes the key differences in children’s oral health legislation and preventive frameworks between Germany and Saudi Arabia.

#### 3.4.2. Oral Health Monitoring Projects and Working Teams

**Figure 6:**
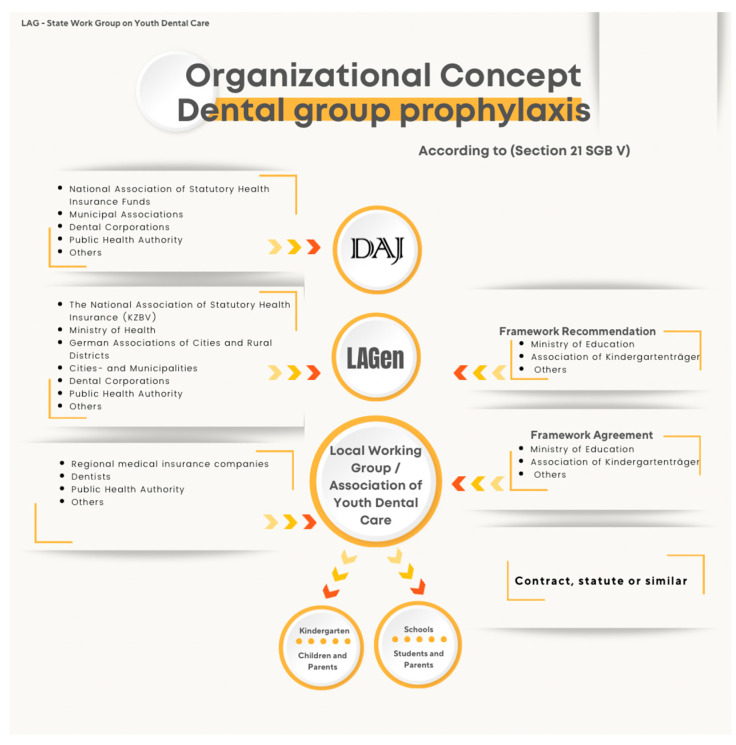
Organizational concept for German dental group prophylaxis according to section 21, SGB V. Source: www.daj.de.

Beyond legislation, school-based oral health systems in both countries rely on monitoring institutions and interdisciplinary working teams. Germany and Saudi Arabia differ in how these monitoring structures are organized. In Germany, a coordinated national framework is led by the German Association for Youth Dental Care (DAJ) and the Institute of German Dentists (IDZ). These institutions define the methodological framework for the German National Oral Health Surveys (DMS), while data collection is carried out by regional working teams in each federal state. Examination procedures follow national guidance, with some regional variation in diagnostic criteria and calibration practices. In addition to the DMS, several specialized epidemiological initiatives—including SHIP [46]; Gekokids [47]; KiGGS [48]; Gesunde Zähne ein Leben lang [49]; IPKiSuN, MundZaRR [50], MuMi [51], MundPflege, InSEMaP and EFAFU [52], collect oral-health related data across different age groups and regions. The Robert Koch Institute (RKI), as the national public health authority, includes oral health indicators in federal health reporting. The organizational structure of the DAJ regional working teams is shown in Figure 6.

**Figure 7:**
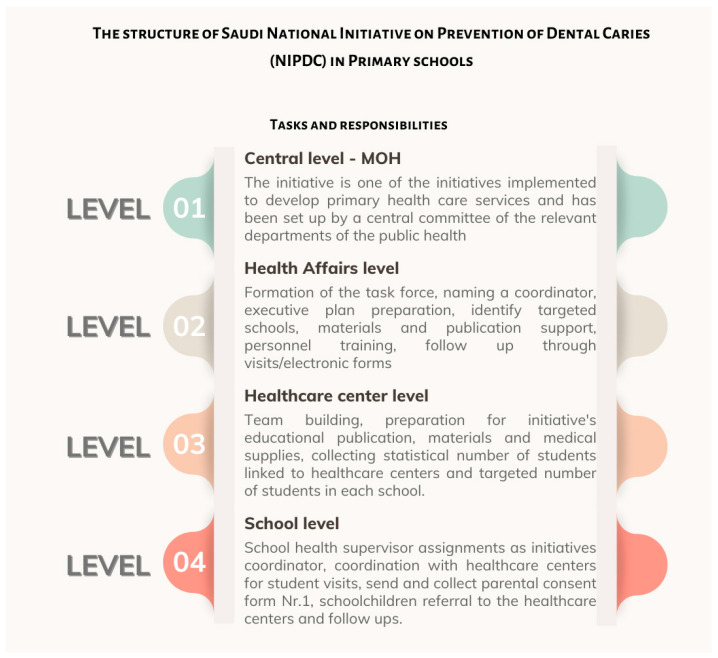
Structure of the Saudi National Initiative for the Prevention of Dental Caries in Primary Schools, outlining tasks across MOH, regional health affairs, health centers, and schools. Source: Presentation by Dr. Saeed M. S. Almohammadi (in Arabic) [53].

In Saudi Arabia, oral health monitoring activities are conducted through regional School Health teams and Primary Health Care Centers. The National Initiative for Preventing Dental Caries (NIPDC) outlines the responsibilities across the Ministry of Health, regional Health Affairs, Primary Health Care Centers, and schools [44]. Publicly available reports or datasets from NIPDC are not currently accessible. The operational structure of NIPDC, as presented in internal Ministry of Health materials, is illustrated in Figure 7.

To provide a structured comparison of national child oral health projects and their monitoring tools, Table 2 contrasts the DAJ- and IDZ-supported projects in Germany with the NIPDC framework in Saudi Arabia across key domains such as surveillance, data standards, dissemination, and project continuity.

#### 3.4.3. Calibration of Examiners

Germany and Saudi Arabia differ in the standardization and transparency of examiner calibration within child oral-health programs. Germany implements structured calibration protocols, with DAJ reporting inter-examiner agreement (κ ≥ 0.65) among 482 calibrated dentists. According to DAJ (2016), a total of 482 dentists underwent online calibration for dental screening [11]. These reliability scores are reported in the DAJ calibration data and are used within Germany’s national oral health surveillance framework [33,34].

By contrast, Saudi Arabia’s National Initiative for the Prevention of Dental Caries (NIPDC) has no publicly available documentation describing examiner calibration protocols. Despite its nationwide implementation, there is no evidence of standardized training, reliability assessments, or formal inter-examiner calibration within the available sources. This absence is documented in the available sources, and no information is provided on whether diagnostic practices are applied consistently across regions, although dmft/DMFT examinations are generally robust.

#### 3.4.4. Data Management

Germany and Saudi Arabia differ in the maturity, transparency, and structure of their oral health data management systems. Germany operates a digitally integrated infrastructure that supports standardized data entry, cross-regional comparability, and routine evaluation. Dental services across federal states employ established software platforms—such as ISGA (InformationsSystem GesundheitsAmt, Computer Zentrum Strausberg GmbH, Strausberg, Germany), Gudental (software.house informationstechnik AG. 24105 Kiel, Germany), Micropro (Mikroprojekt GmbH, Kaiserslautern, Germany), and Octoware^®^NT (easy-soft GmbH, Dresden, Germany)—which enable the systematic recording of demographic variables, examination findings, and program-level indicators. This digital infrastructure also supports longitudinal linkage with national surveys (e.g., DAJ) and facilitates dissemination through regular public reporting [11]. Conversely, Saudi Arabia’s National Initiative for the Prevention of Dental Caries (NIPDC) has no publicly available information describing its data management protocols, including software systems, integration platforms, or standardized recording procedures. Despite formal inquiries, no documentation has been disclosed regarding data flow, quality-control mechanisms, or interoperability across regions. This absence of publicly available documentation means that information on data completeness, program performance, or regional comparability cannot be assessed from the available sources, limiting the ability to compare outcomes across regions. The lack of publicly accessible datasets also precludes trend analysis, dissemination, and assessment of goal attainment.

## 4. Discussion

This section synthesizes the key findings from the comparative analysis of Germany and Saudi Arabia’s children’s oral health and integrates contextual information that supports interpretation of the epidemiological results. By combining caries trends, legal frameworks, operational structures, and governance mechanisms, the discussion highlights how systemic organization shapes preventive coverage, data quality, and long-term outcomes [54].

### 4.1. Caries Epidemiology

Global oral health surveillance began in the late 1960s under the World Health Organization (WHO) leadership, and early international mapping revealed marked disparities in childhood caries between industrialized and developing countries [9].

In Germany, caries monitoring was formally introduced in the 1970s through participation in WHO’s International Collaborative Study (ICS I, 1973–1979), which documented elevated DMFT scores among adolescents [6,55]. Subsequent national surveys have tracked a steady decline in caries experience, with recent data indicating a 90% reduction over the past 45 years [56]. This sustained improvement may be associated with Germany’s legally mandated preventive programs in group and individual settings, standardized diagnostic criteria, calibrated examiners and continuous long-term surveillance [5,57,58]. Despite slight methodological variations between institutions, DMFT remains a reliable and comparable indicator across sources, reinforcing the robustness of Germany’s epidemiological credibility [40].

In contrast, Saudi Arabia faces persistent challenges in oral health data collection and has not participated in global initiatives like WHO ICS. Although some studies report declining dmft scores over the past 10–15 years [39,40,41,42], many combine broad age ranges, potentially confounding age-specific trends. Other investigations—such as Al-Banyan et al. (2000) and Al-Rafee et al. (2019)—continue to report alarmingly high caries prevalence rates in children and adolescents [59,60]. These inconsistencies reflect the absence of calibrated national surveys and highlight the need for a cohesive oral health strategy that establishes reliable baseline indicators, enabling trend analysis, and guiding evidence-based prevention efforts in Saudi Arabia.

### 4.2. Legal Mapping

Legal mapping provides a framework to understand how preventive responsibilities are distributed across institutions and how policies evolved over time [61]. In Germany, preventive dental care is a statutory entitlement enshrined in the Social Code Book V (§21 SGB V). This legal foundation ensures consistent service delivery, regular evaluation, and public accountability. Institutions such as the Federal Joint Committee (G-BA) and the German Association for Youth Dental Care (DAJ) operate under clearly defined mandates, supported by transparent scientific methods and rigorous quality control [11].

In contrast, Saudi Arabia’s legal–epidemiological framework remains underdeveloped. According to Al-Ansari et al. (2019), “no government body is assigned the task of collecting oral health data and in most cases, information about caries is available through published scientific publications without national oral health surveys; mostly due to logistic difficulties than lack of resources [62]”. This finding aligns with Quadri et al. (2018), who observed that supervision of school oral health activities and regular monitoring visits appear limited or insufficiently structured [63].

Saudi Arabia’s Vision 2030 offers a strategic opportunity to strengthen oral health governance through national capacity-building and enhanced institutional transparency. The Vision 2030 Achievements Booklet reports a 65% increase in healthcare professionals between 2016 and 2020, reflecting substantial investment in workforce development. Aldossary (2022) emphasizes Vision 2030’s supportive foundation for healthcare system advancement [64], while Mani and Goniewicz (2024) highlight its transformative impact on governance, digital health, and systemic reform [65].

Despite this progress, establishing a legal basis for school-based oral health—including clearly defined roles, national data mandates, and sustained funding—remains critical. Without legislative codification, programs like the National Initiative for the Prevention of Dental Caries (NIPDC) risk fragmentation, regional inconsistency, and limited accountability. This concern is echoed in recent implementation research on the Child Oral Health Initiative, which underscores how policy innovation and systemic coordination were essential to overcoming fragmented service delivery and achieving preventive care goals [66].

### 4.3. Operational Structures and Monitoring Capacity

Germany’s operational model is characterized by a coordinated national surveillance ecosystem. DAJ and IDZ provide methodological guidance for national oral health surveys (DMS), while regional working teams conduct standardized examinations. Although minor regional variations exist, Germany benefits from harmonized diagnostic criteria (BZÖG), calibrated examiners, and a long-standing research infrastructure that supports longitudinal monitoring.

Saudi Arabia’s monitoring capacity remains fragmented. School Health teams and Primary Health Care Centers conduct screenings without a unified national surveillance system, leading to substantial variation in diagnostic criteria, examiner calibration, and reporting pathways. Where examiner calibration is not standardized, inter-examiner variability may increase and contribute to regional differences in diagnostic consistency; by contrast, Germany’s calibrated workforce supports more reliable and comparable surveillance data. The NIPDC represents the most structured national attempt to coordinate preventive activities, but the absence of publicly available reports, datasets, or methodological documentation limits evaluation of its effectiveness. The lack of centralized coordination and longitudinal surveillance restricts the ability to monitor national trends, assess program performance, or align preventive strategies with international benchmarks.

### 4.4. Data Management and Digital Infrastructure

Germany operates a digitally integrated data infrastructure that supports standardized data entry, cross-regional comparability, and timely evaluation. Software platforms such as ISGA, Gudental, Micropro, and Octoware/Easy-Soft enable systematic recording of demographic variables, examination findings, and program indicators [11]. This digital ecosystem minimizes documentation errors, facilitates linkage with national surveys, and supports regular public reporting, thereby strengthening the validity and comparability of national surveillance outputs.

Conversely, Saudi Arabia’s NIPDC lacks publicly available information on data-management protocols, software systems, or quality-control mechanisms. Despite formal inquiries, no documentation has been disclosed regarding data flow, interoperability, or regional standardization. This absence of transparency limits the ability to assess data completeness, evaluate program performance, compare outcomes across regions, or conduct trend analysis—a component essential for evidence-based policy development.

### 4.5. Strengths and Limitations

This study presents several strengths, notably offering the first comparative review of legal frameworks underpinning the implementation of school-based oral health policies in Germany and Saudi Arabia. It delivers a structured assessment of prevention strategies, supported by the integration of previously unpublished insights from Saudi health and education authorities. Notable contributions include

- Contextual observations on service outreach and school health unit involvement in local school-health programs;
- Observational insights on oral health program distribution at the local level;
- Comparative analysis integrating national surveillance benchmarks from Germany.

Such inclusions strengthen the study’s empirical contribution and provide fresh insight into Saudi Arabia’s evolving oral health landscape.

However, limitations must be acknowledged. In Saudi Arabia, data accuracy on caries prevalence and prevention efforts is challenged by fragmented reporting, lack of calibrated examiners, and inconsistent digital infrastructure—a concern documented in national implementation studies [23]. These methodological disparities contrast with Germany’s calibrated workforce, standardized electronic surveillance systems and widespread mandatory oral examinations [67]. Additionally, variability in sample sizes and age classification—such as first-grade cohorts in Saudi Arabia ranging from 5 to 8 years old—affects cross-national comparability and limits age-specific analysis.

Despite these constraints, the study identifies a consistently higher caries burden in Saudi Arabia, underscoring the need for improved surveillance, legal codification, and targeted prevention strategies. While data asymmetry precludes direct statistical comparison, the structural analysis may offer valuable guidance for future policy refinement and system-building efforts.

### 4.6. Suggestions

The absence of a nationally coordinated children’s oral health strategy in Saudi Arabia appears to have contributed to fragmented service delivery and inconsistent monitoring across sectors. Limited integration between key ministries and providers—particularly the Ministry of Health and the Ministry of Education—may weaken the implementation and sustainability of preventive dental initiatives for children. Current school-based programs vary in scope, reach, and follow-up mechanisms, which may contribute to unequal access and persistent gaps in oral health outcomes.

Drawing on international benchmarks, valuable insights can be extracted from Germany’s centralized oral health system. The German model, implemented through the German Association for Youth Dental Care (DAJ e.V.) and supported by national legislation, is cohesive and unified, helping to ensure more equitable access to preventive dental care for children, irrespective of school sector or governing authority. This structure supports consistent service delivery, standardized monitoring, and better nationwide health equity. In contrast, Saudi Arabia’s oral health framework remains sectoral and segmented, with fragmented delivery mechanisms and parallel programs overseen by different ministries and providers. This decentralized structure may underlie the observable gaps and inconsistencies in service coverage and evaluation processes, limiting the reach and long-term impact of school-based initiatives. Integrating lessons from the German model may offer a strategic opportunity for Saudi policymakers to develop a more coordinated and equitable oral health framework. Emphasizing national alignment, legislative support, and inter-ministerial integration could help pave the way for a sustainable system that improves access to preventive care for all schoolchildren. It may be assumed that integrated data governance within pediatric dental programs enables cross-sectoral collaboration, improves transparency, and supports value-based health financing models in oral health. This hypothesis is supported by findings from Germany, where coordinated data-entry protocols across school dental programs enabled consistent examination standards and centralized access to oral health indicators. Consequently, a proposed pilot in Qassim could replicate this by implementing structured examination workflows, digitizing oral-examinations records, and building an intersectoral oral-health coalition. This model would foster transparency, accelerate evidence-based preventive policy formulation, and strengthen accountability across providers, educators, and policymakers.

**Figure 8:**
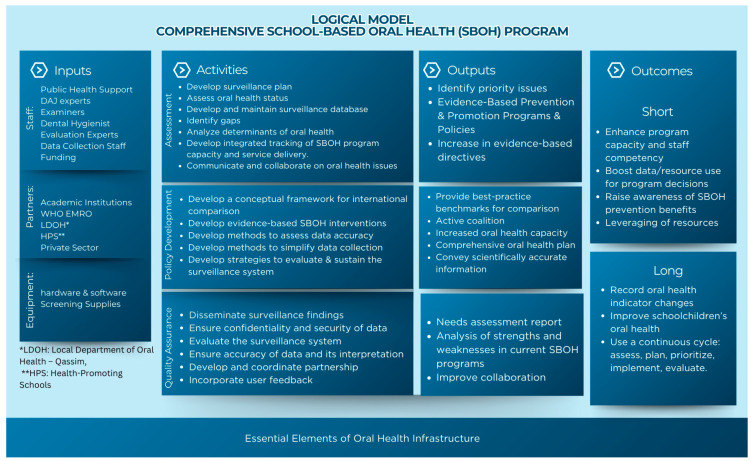
Proposed logic model for a comprehensive Saudi School-based oral health program (modified framework).

Reforms must be evidence-based and contextually tailored to Saudi Arabia’s governance structures and social norms. Priorities include formalizing inter-ministerial roles, instituting standardized surveillance protocols, and adopting secure digital tools for routine screening and reporting. Figure 8 consolidates these recommendations into a single logic model that links inputs and activities to measurable outputs and short- and long-term outcomes, offering a practical blueprint for phased implementation and policy evaluation.

## 5. Conclusions

This comparative analysis underscores that the systemic structure, not merely the existence of services, is a pivotal determinant of caries prevalence among children. Germany’s success stems from its legally mandated age-specific oral health programs, calibrated workforce, and a unified data strategy. These elements collectively ensure accountability, equity, and measurable outcomes. By contrast, Saudi Arabia’s fragmented framework results in diagnostic variability, uneven implementation, and limited policy traction despite considerable investment.

It appears that the means for transition from scattered initiatives to sustained impact are present in Saudi Arabia, but oral health should be embedded into national legislation, and interoperable digital surveillance platforms should be made available for regular examination by calibrated examiners. These reforms should be augmented by strategic coalitions and evidence-informed interventions across regions. Well-resourced areas such as Qassim can serve as innovation hubs to pilot scalable programs, implement AI-integrated school–health models, and support nonprofit research centers that inform national policy.

Inspired by international best practices—as Germany itself learned from Scandinavian prevention models—the path forward requires more than replication. It calls for adaptation that respects local contexts while aligning with global benchmarks. Future research should not only fill epidemiological gaps but also rigorously assess structural reforms. Only through such systemic re-engineering can Saudi Arabia progress toward equitable, preventive oral health care for every child.

## Figures and Tables

**Table 1:** Comparison of children’s oral health legislation and preventive frameworks in Germany and Saudi Arabia.

Domain	Germany	Saudi Arabia
Legal foundation	Legally mandated preventive services under Social Code Book V (SGB V)	No unified legal mandate for children’s oral health
Preventive structure	Structured, age-specific entitlements (GP, IP, FU exams)	Preventive services delivered through initiative-based programs (SDPP, NIPDC)
National surveillance	National Oral Health Surveys (DAJ/IDZ) with standardized monitoring	No national oral-health survey; reliance on regional or program-based data
Implementation consistency	Uniform nationwide implementation through statutory health insurance	Variable implementation depending on regional capacity and program availability
Early childhood prevention	Mandated early detection exams from 6 months; fluoride varnish and caregiver education	Preventive guidelines exist, but implementation is not legally mandated ^1^
Integration with medical care	Coordinated documentation (CDB, Yellow Booklets) linking dentists and pediatrician	Limited integration; preventive guidance delivered mainly through MOH programs
Governance	Strong regulatory oversight with standardized examiner calibration	Decentralized implementation with limited calibration and monitoring

^1^ The Saudi Clinical Preventive Guideline (2023) issued by the Public Health Authority.

**Table 2:** Comparison of national child oral health project structures and monitoring frameworks in Germany and Saudi Arabia.

Domain	Germany	Saudi Arabia
Project initiation	DAJ established 1994/95; DMS cycles since 1989; long-term institutional continuity	NIPDC launched 2017; initiative-based rather than institutionalized
Public-health integration	Oral health indicators integrated into federal health reporting via RKI	No national mechanism for integrating oral health indicators
Working team structure	Coordinated DAJ regional teams; federal model with harmonized but not uniform criteria	NIPDC structure linking MOH → Regional Health Affairs → PHCs → Schools
Diagnostic criteria and calibration	National methodological guidance; regional variation acknowledged	No national diagnostic standardization; calibration practices vary
Data standardization	Harmonized data-collection protocols across federal states	Variable diagnostic practices; no standardized reporting
Dissemination	Regular public reports, DMS publications, RKI health reporting, peer-reviewed outputs	No public dissemination; no official NIPDC reports or datasets available
Quality assurance	Quality-control mechanisms within DAJ and IDZ	No publicly documented quality-assurance procedures
Trend analysis	Longitudinal datasets available	No national trend analysis possible due to absence of data
Aim achievement/Goal attainment	Improvements in caries indicators documented across DMS cycles	No published evaluation reports available

## Data Availability

All data used in this study are derived from previously published sources, including national oral health surveys, peer reviewed literature, and official reports. All datasets are publicly available as cited in the references. No new or unpublished data were generated or analyzed in this study.
